# Performance-Enhanced Textured Silicon Solar Cells Based on Plasmonic Light Scattering Using Silver and Indium Nanoparticles

**DOI:** 10.3390/ma8105330

**Published:** 2015-09-25

**Authors:** Wen-Jeng Ho, Shih-Ya Su, Yi-Yu Lee, Hong-Jhang Syu, Ching-Fuh Lin

**Affiliations:** 1 Department of Electro-Optical Engineering, National Taipei University of Technology, No. 1, Section 3, Zhongxial East Road, Taipei 10608, Taiwan; susuyu15@gmail.com (S.-Y.S.); t8659009@ntut.edu.tw (Y.-Y.L.); 2 Graduate Institute of Photonics and Optoelectronics, National Taiwan University, No. 1, Section 4, Roosevelt Road, Taipei 10617, Taiwan; f98941054@ntu.edu.tw (H.-J.S.); lincf@ntu.edu.tw (C.-F.L.)

**Keywords:** indium nanoparticles (In-NPs), plasmonic light scattering, silver nanoparticles (Ag-NPs), textured crystalline-silicon solar cells

## Abstract

Performances of textured crystalline-silicon (c-Si) solar cells enhanced by silver nanoparticles (Ag-NPs) and indium nanoparticles (In-NPs) plasmonic effects are experimentally demonstrated and compared. Plasmonic nanoparticles integrated into textured c-Si solar cells can further increase the absorption and enhance the short-circuit current density (*J*_sc_) of the solar cell. To examine the profile of the proposed metallic particles, the average diameter and coverage of the In-NPs (Ag-NPs) at 17.7 nm (19.07 nm) and 30.5% (35.1%), respectively, were obtained using scanning electron microscopy. Optical reflectance and external quantum efficiency response were used to measure plasmonic light scattering at various wavelengths. Compared to a bare reference cell, the application of In-NPs increased the *J*_sc_ of the cells by 8.64% (from 30.32 to 32.94 mA/cm^2^), whereas the application of Ag-NPs led to an increase of 4.71% (from 30.32 to 31.75 mA/cm^2^). The conversion efficiency of cells with embedded In-NPs (14.85%) exceeded that of cells with embedded Ag-NPs (14.32%), which can be attributed to the broadband plasmonic light scattering of the In-NPs.

## 1. Introduction

Most solar cells are made of silicon-based wafers with a thickness of 150–200 μm. Light trapping in crystalline-silicon (c-Si) solar cells is generally achieved using pyramidal structures on the surface to scatter incident light into the solar cell [1,2,3,4,5]. The efficiency of solar cells is also enhanced by plasmonic light scattering using metallic nanoparticles (NPs) [6,7,8,9]. Incident light excites a localized surface plasmon resonance in metallic nanoparticles, which then couples incident light into the cell over a broad angular range [10,11,12,13]. Metal NPs can be applied to solar cells after cell device processing and separated from the surface of the semiconductor by a dielectric spacer layer. Therefore, it has been predicted that metallic NPs deposited on fabricated textured c-Si solar cells would further enhance light trapping as well as increase their efficiency [14,15]. However, plasmonic light trapping depends on the properties (shape and size) of the metallic particles as well as the surrounding material [16]. Furthermore, embedding metal NPs in different dielectric materials can significantly alter the scattering properties [17,18,19].

In this paper, plasmonic textured c-Si solar cells using a TiO_2_ spacer layer and indium (In) NPs or silver (Ag) NPs are proposed. We then characterized the electrical and optical properties using measurements of reflectance and external quantum efficiency (EQE). The novelty in the plasmonsic effects of In-NPs (with a broadband) and Ag-NPs (with a narrow band and at longer wavelengths) can be clearly seen in the spectrum of reflectivity and EQE. Finally, we investigated the photovoltaic performance of the resulting c-Si solar cells with TiO_2_/In-NPs/Al_2_O_3_ and with TiO_2_/Ag-NPs/Al_2_O_3_ configurations in order to differentiate the plasmonic effects of In-NPs and Ag-NPs.

## 2. Experiment

### 2.1. Fabrication of Textured Crystalline-Silicon (c-Si) Solar Cells

**Figure 1:**
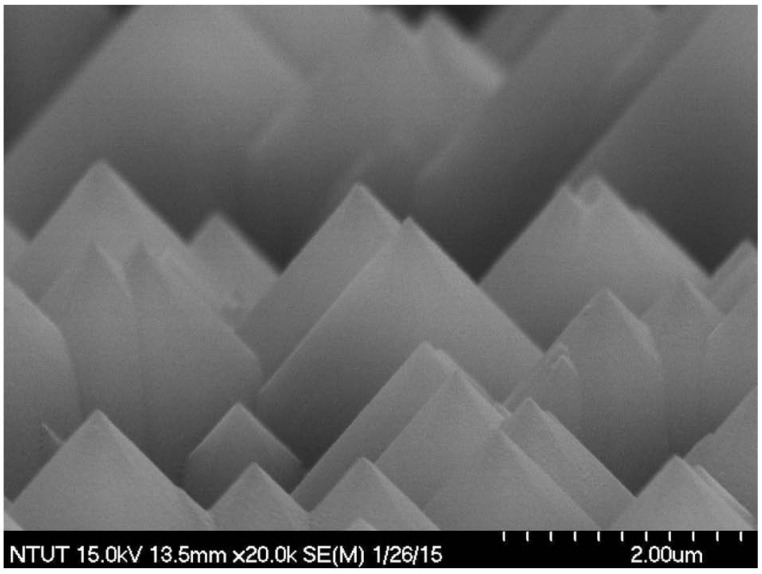
Scanning electron microscopy (SEM) image showing the surface of crystalline-silicon (c-Si) wafer after etching by being dipped into a solution of H_2_O/KOH/IPA (Isopropanol).

A textured c-Si solar cell with a thickness of 180-μm thick was fabricated using the following process. The saw-damaged surface of boron doped c-Si wafer was removed by dipping the wafer in a H_2_O/KOH (Potassium) solution. The surface of the wafer was then etched by dipping in a solution of H_2_O/KOH/IPA (Isopropanol). The resulting textured surface was then examined using scanning electron microscopy (SEM; Hitachi S-4700, Hitachi High-Tech Fielding Corporation, Tokyo, Japan), as shown in Figure 1. After RCA (Radio Corporation of America) cleaning, an n^+^-Si emitter layer with a sheet resistance of approximately 80 Ω/sq was applied to the textured wafer using a POCl_3_ diffusion process in a tube diffusion chamber at 850 °C for 3 min. The oxide layer on the surface of the wafer was removed using hydrogen fluoride (HF) solution before an Al film was deposited on the rear surface using electron-beam (e-beam) evaporation. The wafer was then annealed at 450 °C for 5 min to form the back electrode. Finally, top contact grid-electrodes with a 20 nm Ti film and 300 nm Al film were formed using photolithography lift-off and e-beam evaporation, which resulted in bare textured c-Si solar cells. The surface properties of the textured c-Si were characterized using SEM and optical reflectance measurement (Lambda 35, PerkinElmer, Inc., Waltham, MA, USA); the photovoltaic performance was determined according to photovoltaic current-voltage (I-V) under air mass (AM) 1.5 G illumination. The solar simulator (XES-151S, San-Ei Electric Co., Ltd., Osaka, Japan) was calibrated using a National Renewable Energy Laboratory (NREL)-certified crystalline silicon reference (PVM-236) prior to measurement. The bare textured cells having approximately the same device performance, such as short-circuit current (*I*_sc_) and conversion efficiency (η), are selected firstly. The selected cells are a baseline for further device processing and subsequently performance measurement.

### 2.2. Characterization of Textured c-Si Solar Cells with In-NPs and Ag-NPs

**Figure 2:**
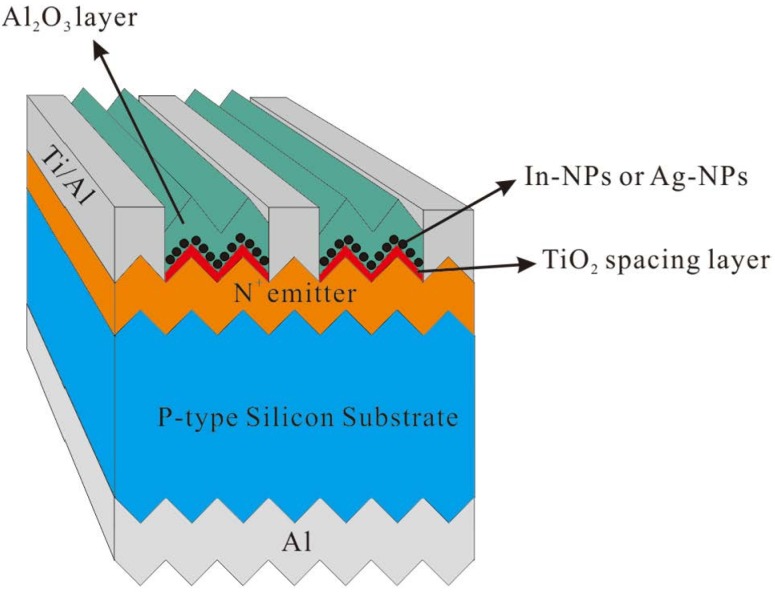
Schematic diagram of textured silicon solar cells with the following configurations: TiO_2_/indium nanoparticles (In-NPs)/Al_2_O_3_ and TiO_2_/silver nanoparticles (Ag-NPs)/Al_2_O_3_.

To investigate the plasmonic effects of metallic NPs on the textured c-Si solar cells, we began by depositing a 15-nm thick TiO_2_ spacer layer on the bare textured c-Si solar cells using e-beam evaporation. Using e-beam evaporation, a 3.8-nm thick film of indium (In) was then deposited over the TiO_2_ spacer layer of one cell while a 4.5-nm thick film of silver (Ag) was deposited over the TiO_2_ spacer layer of another cell. The cells were subsequently annealed in an RTA chamber at 200 °C for 10 min under ambient H_2_ to form In-NPs and Ag-NPs. According to previous study in our laboratory, In NPs with a 3.8-nm thick In-film presented the best performances rather than that of with the thickness of 2.5, 5.2 and 7.1 nm. Therefore, we attempted to control the dimensions and coverage of these metallic particles as closely as possible to facilitate the comparison of the plasmonic effects of In-NPs and Ag-NPs. Finally, we deposited a 65-nm thick film of Al_2_O_3_ over the NPs to reveal the influence of a surrounding material on plasmonic light scattering. Figure 2 presents a schematic diagram of the plasmonic textured silicon solar cells consisting of the following structures: TiO_2_/In-NPs/Al_2_O_3_ and TiO_2_/Ag-NPs/Al_2_O_3_. We then compared the optical reflectance, external quantum efficiency (EQE, Enli Technology Co., Ltd., Kaohsiung, Taiwan), and photovoltaic performance of cells with and without metallic NPs as well as cells with and without a layer of Al_2_O_3_ over the metallic NPs. The difference between In-NPs and Ag-NPs with regard to plasmonic light scattering was determined according to EQE response at various wavelength ranges. In addition, we measured the improvements in photovoltaic performance achieved by the application of In-NPs or Ag-NPs, compared to a reference solar cell.

## 3. Results and Discussion

**Figure 3:**
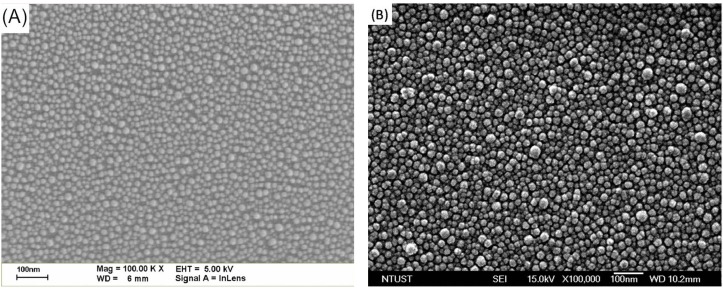
SEM images showing the size and profile of metallic nanoparticles: (**A**) In-NPs; (**B**) Ag-NPs.

**Figure 4:**
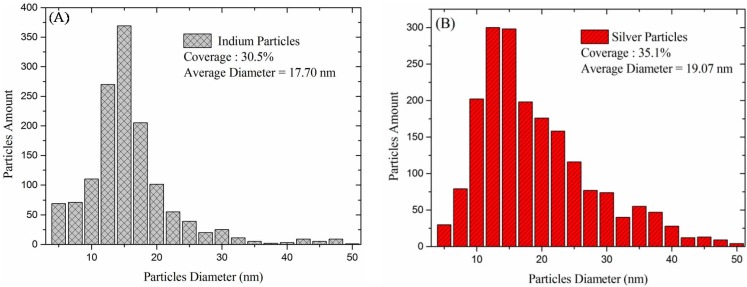
Particle diameter distribution and coverage: (**A**) In-NPs and (**B**) Ag-NPs. These values were calculated by analyzing the SEM images in Figure 3A,B using J-image software.

**Figure 5:**
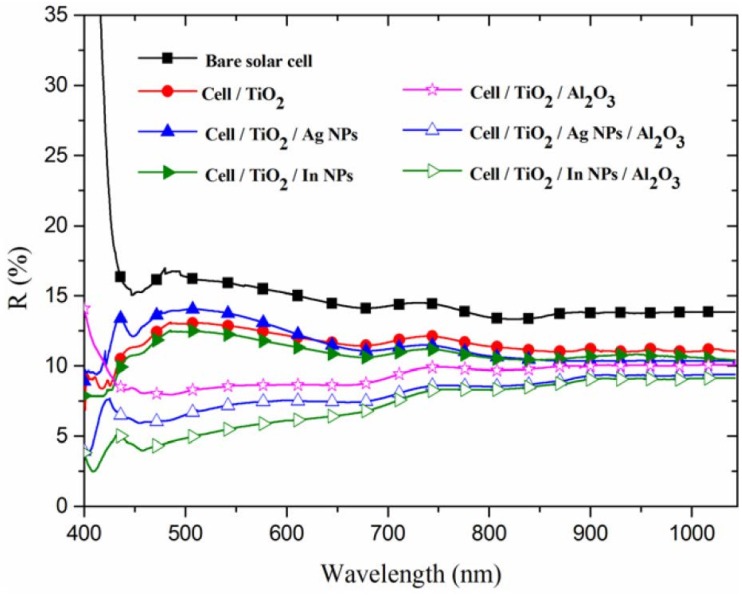
Optical reflectance (*R*) of bare textured c-Si solar cell, cell with TiO_2_ spacer layer, cell with double layer of TiO_2_/Al_2_O_3_, cell with In-NPs on TiO_2_ spacer layer, cell with Ag-NPs on TiO_2_ spacer layer, cell with In-NPs embedded in TiO_2_/Al_2_O_3_, and cell with Ag-NPs embedded in TiO_2_/Al_2_O_3_.

SEM was used to determine the size and profile of the In-NPs and Ag-NPs, as shown in Figure 3A,B, respectively. As shown in Figure 4A, the average diameter and coverage of the In-NPs were 17.7 nm and 30.5%, respectively. As shown in Figure 4B, the average diameter and coverage of the Ag-NPs were 19.07 nm and 35.1%. These values were calculated by analyzing the SEM images in Figure 3A,B using J-image software (National Institute of Mental Health, Bethesda, MD, USA). The SEM results show that the dimensions and coverage of these metallic particles were controlled as closely as possible to facilitate the comparison of the plasmonic effects of In-NPs and Ag-NPs. Figure 5 presents the optical reflectance of (1) bare textured c-Si solar cells; (2) a cell coated with a TiO_2_ spacer layer; (3) a cell coated with a double layer of TiO_2_/Al_2_O_3_; (4) a cell with In and Ag-NPs deposited over TiO_2_ spacer layer; and (5) a cell with In and Ag-NPs embedded in layer of TiO_2_/Al_2_O_3_. The reflectance of the cells coated with a layer of TiO_2_ or a double layer of TiO_2_/Al_2_O_3_ was lower than that of the bare cell. As In-NPs formed upon the TiO_2_ spacer layer, the reflectance decreased to a level lower than that of the cell with TiO_2_ in full wavelength range, due to broadband plasmonic scattering of In-NPs. Notably, the reflectance of the cell with Ag-NPs/TiO_2_/c-Si exceeded that of the cell with TiO_2_/c-Si at wavelengths below 620 nm due to optical reflection of Ag-NPs; however, it was lower at wavelengths beyond 620 nm due to plasmonic scattering of Ag-NPs. Differences in the plasmonsic effects of In-NPs (with a broadband) and Ag-NPs (with a narrow band and at longer wavelengths) on the Si can be clearly seen in the spectrum of reflectivity. As shown in Figure 5, the overall reflectivity of the In-NP/TiO_2_/c-Si was far below that of Ag-NP/TiO_2_/c-Si. The application of the Al_2_O_3_ layer over the NPs further reduced reflectivity to below that of the cell with Al_2_O_3_/TiO_2_/c-Si, particularly at wavelengths below 700 nm due to antireflection of Al_2_O_3_ layer and plasmonic scattering of the metallic NPs.

**Figure 6:**
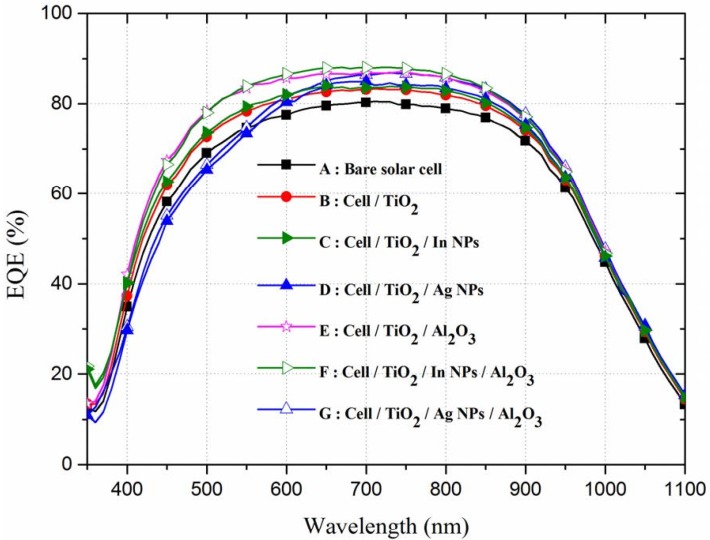
External quantum efficiency (EQE) response of bare textured c-Si solar cell, cell with TiO_2_ spacer layer, cell with double layer of TiO_2_/Al_2_O_3_, cell with In-NPs on TiO_2_ spacer layer, cell with Ag-NPs on TiO_2_ spacer layer, cell with In-NPs embedded in TiO_2_/Al_2_O_3_, and cell with Ag-NPs embedded in TiO_2_/Al_2_O_3_.

**Figure 7:**
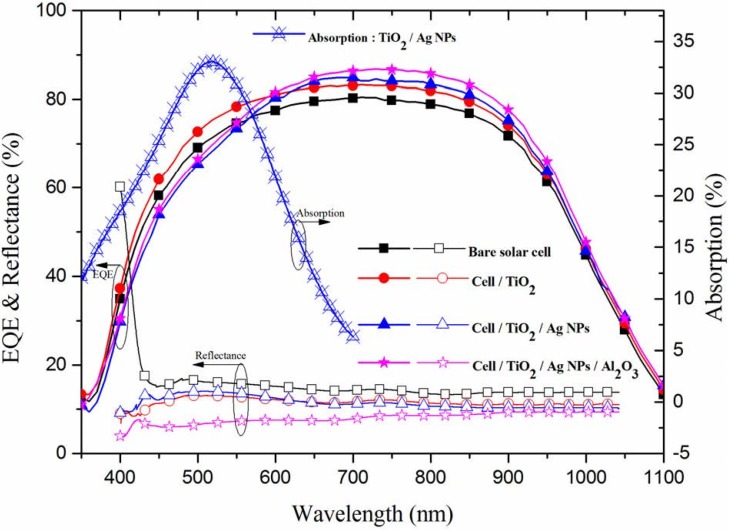
EQE response and optical reflectance of bare textured c-Si solar cell, cell with TiO_2_ spacer layer, cell with Ag-NPs on TiO_2_ spacer layer, and cell with Ag-NPs embedded in TiO_2_/Al_2_O_3_.

Figure 6 presents the EQE response of all of the cells evaluated in this study. Coating the cells with a TiO_2_ spacer layer or a double layer of TiO_2_/Al_2_O_3_ increased the EQE values of the cell at wavelengths from 350 to 1100 nm compared to the bare solar cell, which is in agreement with the results of optical reflectance. Across the full range of wavelengths, the cell with In-NPs presented higher EQE values than did the cell with only a TiO_2_ layer, which is also in agreement with the results of optical reflectance due to broadband plasmonic scattering of In-NPs. The cell with Ag-NPs on the TiO_2_ spacer layer presented lower EQE values than did the bare cell at wavelength below 550 nm, which is not in agreement with the results of optical reflectance. The lower EQE values at shorter wavelengths (<550 nm), as shown in Figure 7, can be attributed to the incident photons absorbing by Ag-NPs [20,21]. The absorption (α) of photons in the Ag-NPs/TiO_2_/glass was calculated as follows:

α = 1 − *R* − *T* (1)

where *R* is the measured reflectance and *T* is the measured transmittance of the proposed sample. As shown in Figure 6, the overall EQE response of the cells with Al_2_O_3_ deposited over In-NPs was superior to that of the cell with Al_2_O_3_ deposited over Ag-NPs because of the broadband plasmonic scattering of In-NPs.

**Figure 8:**
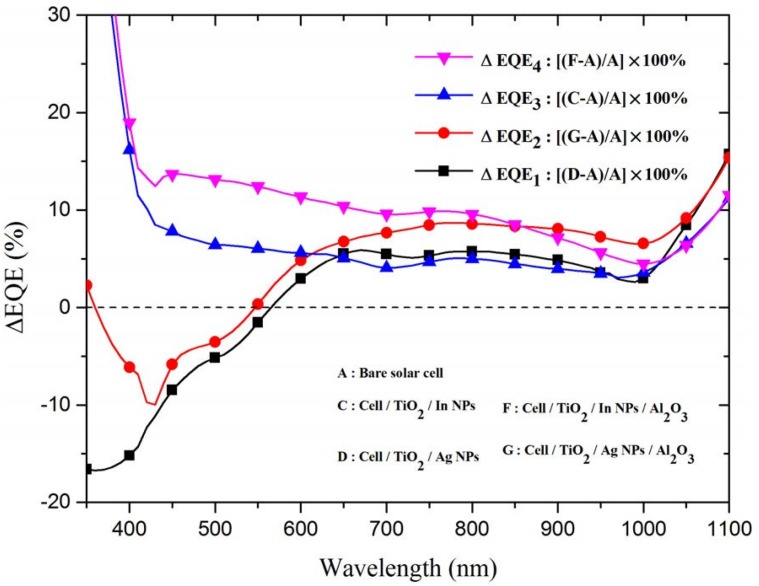
EQE enhancements (ΔEQE) of cell with In-NPs on TiO_2_ spacer layer, cell with Ag-NPs on TiO_2_ spacer layer, cell with In-NPs embedded in the TiO_2_/Al_2_O_3_, and cell with Ag-NPs embedded in TiO_2_/Al_2_O_3_.

Figure 8 presents the improvements in EQE (ΔEQE) of all cells evaluated in these experiments. The ΔEQE of the cell with In-NPs exceeded that of the cell with Ag-NPs over but not at wavelengths beyond 650 nm. Because the incident photons are absorbed by Ag-NPs at a wavelength range of 350–650 nm and had a higher plasmonic scattering than In-NPs at wavelengths beyond 650 nm. Furthermore, the ΔEQE of the cell with In-NPs embedded in TiO_2_/Al_2_O_3_ also exceeded that of the cell with Ag-NPs embedded in TiO_2_/Al_2_O_3_ over a wavelength range of 350–850 nm, due to the contribution of the antireflection of Al_2_O_3_ layer and plasmonic scattering of the metallic NPs.

**Figure 9:**
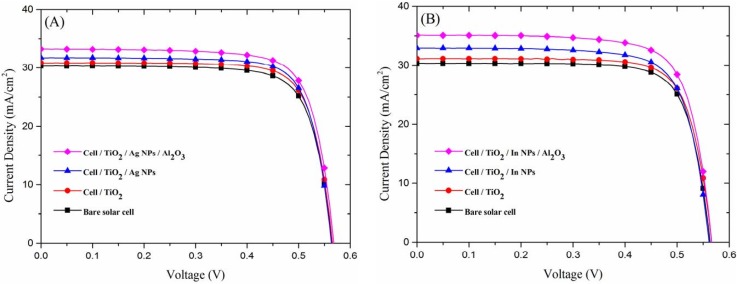
Photovoltaic current density-voltage (J-V) curves: (**A**) Bare cell, cell with TiO_2_ spacer layer, cell with Ag-NPs on TiO_2_ spacer layer, and cell with Ag-NPs embedded in Al_2_O_3_; (**B**) Bare cell, cell with TiO_2_ spacer layer, cell with In-NPs on TiO_2_ spacer layer, and cell with In-NPs embedded in Al_2_O_3_.

Figure 9A presents the typical photovoltaic current density-voltage (J-V) curves of the bare cell, the cell with a TiO_2_ spacer layer, the cell with Ag-NPs, and the cell with Ag-NPs embedded in Al_2_O_3_ (the cell-4 marked with “*” in Table 1). The photovoltaic-performance statistics data of four cells cell with and without Ag-NPs were showed in Table 1. Figure 9B presents the photovoltaic current density-voltage (J-V) curves of the bare cell, the cell with a TiO_2_ spacer layer, the cell with In-NPs, and the cell with In-NPs embedded in Al_2_O_3_ (the cell-1 marked with “*” in Table 2). The photovoltaic-performance statistics data of three cells cell with and without In-NPs were showed in Table 2. The mark “*” in Table 1 and Table 2 was the selected bare-type cell having the same of 30.32 mA/cm^2^ for further comparing. In this study, J-V data were measured at etch device processing stages from the bare cell, cell with a TiO_2_ spacer layer, cell with In-NPs (or Ag-NPs) on TiO_2_ spacer layer, to cell with In-NPs (or Ag-NPs) embedded in TiO_2_/Al_2_O_3_ layer. The application of In-NPs/TiO_2_ increased the short-circuit current density (*J*_sc_) of the cells by 8.64% (from 30.32 to 32.94 mA/cm^2^), whereas the application of Ag-NPs/TiO_2_ led to an increase of only 4.71% (from 30.32 to 31.75 mA/cm^2^). Embedding In-NPs in TiO_2_/Al_2_O_3_ increased the short-circuit current density (*J*_sc_) of the cells by 15.73% (from 30.32 to 35.09 mA/cm^2^), whereas embedding Ag-NPs in TiO_2_/Al_2_O_3_ led to an increase of only 9.73% (from 30.32 to 33.27 mA/cm^2^), due to the contribution of the antireflection of Al_2_O_3_ layer and plasmonic scattering of the metallic NPs. The increase in *J*_sc_ is in good agreement with the EQE responses. The In-NPs had a greater effect on *J*_sc_ than did the Ag-NPs due to their broadband EQE response. Thus, the conversion efficiency of the textured c-Si cell with TiO_2_/In-NPs/Al_2_O_3_ configuration (increased from 13.14% to 14.85%) was better than that of the textured c-Si cell with TiO_2_/Ag-NPs/Al_2_O_3_ configuration (increased from 13.07% to 14.32%), compared to the reference cell, respectively.

**Table 1:** Photovoltaic performance of four bare cells, cells with a TiO_2_ spacer layer, cells with Ag-NPs, and cells with Ag-NPs embedded in Al_2_O_3_.

Parameters	Cell #	Bare Solar Cell	Cell/TiO_2_	Cell/TiO_2_/Ag-NPs	Cell/TiO_2_/Ag-NPs/Al_2_O_3_
*V*_oc_ (mV)	Cell-1	562.5	564.4	561.8	565.5
	Cell-2	567.1	566.8	563.7	566.5
	Cell-3	565.9	566.0	563.4	567.8
	Cell-4 *	564.9	565.6	563.5	568.4
	Average	565.1	565.7	563.1	567.1
*J*_sc_ (mA/cm^2^)	Cell-1	30.11	30.45	30.64	32.41
	Cell-2	30.16	30.65	31.25	33.26
	Cell-3	30.12	30.89	32.51	33.04
	Cell-4 *	30.32	30.86	31.75	33.27
	Average	30.18	30.71	31.54	33.00
η (%)	Cell-1	12.71	12.91	13.10	13.63
	Cell-2	13.17	13.30	13.36	13.73
	Cell-3	13.18	13.50	13.80	14.08
	Cell-4 *	13.07	13.50	13.82	14.32
	Average	13.03	13.30	13.52	13.94

Note: Four individual cells are fabricated on a processing substrate. *: The cell was selected due to the same of *J*_sc_ (30.32 mA/cm^2^) in bare-type for further comparing. *J*_sc_: Short-circuit current density. *V*_oc_: Open-circuit voltage. η: Conversion efficiency.

**Table 2:** Photovoltaic performance of three bare cells, cells with a TiO_2_ spacer layer, cells with In-NPs, and cells with In-NPs embedded in Al_2_O_3_.

Parameters	Cell #	Bare Solar Cell	Cell/TiO_2_	Cell/TiO_2_/In-NPs	Cell/TiO_2_/In-NPs/Al_2_O_3_
*V*_oc_ (mV)	Cell-1 *	563.3	566.4	561.3	566.6
	Cell-2	564.9	566.4	564.1	567.3
	Cell-3	564.6	566.0	564.2	567.5
	Cell-4	X	X	X	X
	Average	564.3	566.3	563.2	567.1
*J*_sc_ (mA/cm^2^)	Cell-1 *	30.32	31.14	32.94	35.09
	Cell-2	30.25	31.26	32.74	35.14
	Cell-3	30.48	31.20	32.43	35.20
	Cell-4	X	X	X	X
	Average	30.35	31.20	32.70	35.14
η (%)	Cell-1 *	13.14	13.54	13.84	13.85
	Cell-2	13.18	13.39	14.14	14.87
	Cell-3	13.35	13.55	14.18	15.00
	Cell-4	X	X	X	X
	Average	13.22	13.49	14.05	14.57

Note: Four individual cells are fabricated on a processing substrate. *: The cell was selected due to the same of *J*_sc_ (30.32 mA/cm^2^) in bare-type for further comparing. X: The cell was failed during device processing.

## 4. Conclusions

This study fabricated plasmonic textured crystalline silicon solar cells with the following configurations: TiO_2_/In-NPs/Al_2_O_3_ and TiO_2_/Ag-NPs/Al_2_O_3_. We then examined the optical reflectance, EQE response, and photovoltaic performance of the resulting cells in order to determine the plasmonic effects of the two NPs. The EQE results indicated that the impressive *J*_sc_ enhancement of the textured c-Si cell due to plasmonic broadband light scattering was higher for the cell with In-NPs (15.73%) than for that with Ag-NPs (9.73%). The broadband plasmonic effects of the In-NPs led to higher efficiency enhancement of 13.01% (from 13.14% to 14.85%) when embedded in Al_2_O_3_, compared to the 9.56% (from 13.07% to 14.32%) in the cell with embedded Ag-NPs.

## References

[1] Green M.A. (1991). Recent progress in crystalline and polycrys-talline silicon solar cells. Solar Energy Mater..

[2] Dimitrova D.Z., Dua C.H. (2013). Crystalline silicon solar cells with micro/nano texture. Appl. Surf. Sci..

[3] Dimitrov D.Z.C., Lin C.H., Du C.H., Lan C.W. (2011). Nanotextured crystalline silicon solar cells. Phys. Status Solidi A.

[4] Park H., Kwon S., Lee J.S., Lim H.J., Yoon S., Kim D. (2009). Improvement on surface texturing of single crystalline silicon for solar cells by saw-damage etching using an acidic solution. Sol. Energy Mater. Sol. Cells.

[5] Xi Z., Yang D., Dan W., Jun C., Li X., Que D. (2004). Investigation of texturization for monocrystalline silicon solar cells with different kinds of alkaline. Renew. Energy.

[6] Das N.C. (2011). Tunable infrared plasmonic absorption by metallic nanoparticles. J. Appl. Phys..

[7] Foslia C.H., Thøgersena A., Karazhanova S., Marsteina E.S. (2011). Plasmonics for light trapping in silicon solar cells. Energy Procedia.

[8] Fahim N.F., Ouyang Z., Jia B., Zhang Y., Shi Z., Gu M. (2012). Enhanced photocurrent in crystalline silicon solar cells by hybrid plasminic antireflection coating. Appl. Phys. Lett..

[9] Zhang Y., Jia B., Ouyang Z., Gu M. (2014). Influence of rear local silver nanoparticle induced light losses in the light trapping of silicon wafer-based solar cells. J. Appl. Phys..

[10] Anno E., Tianimoto M. (2005). Size-dependent change in interband absorption and broadening of optical plasma-resonance absorption of indium particles. J. Appl. Rhys..

[11] Atwater H.A., Polman A. (2010). Plasmonics for improved photovoltaic devices. Nat. Mater..

[12] Zhang Y.N., Ouyang Z., Stokes N., Jia B.H., Shi Z.R. (2012). Low cost and high performance Al nanoparticles for broadband light trapping in Si wafer solar cells. Appl. Phys. Lett..

[13] Temp T.L., Bagnall D.M. (2013). Broadband scattering of the solar spectrum by spherical metal nanoparticles. Prog. Photovolt. Res. Appl..

[14] Dai H., Li M., Li Y., Yu H., Bai F., Ren X. (2012). Effective light trapping enhancement by plasmonic Ag nanoparticles on silicon pyramid surface. Opt. Express.

[15] Chen X., Jia B., Zhang Y., Gu M. (2013). Exceeding the limit of plasmonic light trapping in textured screen-printed solar cells using Al nanoparticles and wrinkle-like graphene sheets. Light Scie. Appl..

[16] Kelly K.L., Coronado E., Zhao L.L., Schatz G.C. (2003). The optical properties of metal nanoparticles: The influence of size, shape, and dielectric environment. J. Phys. Chem. B.

[17] Kobayashi Y., Correa-Duarte M.A., Liz-Marzán L.M. (2001). Sol-gel processing of silica-coated gold nanoparticles. Langmuir.

[18] Liu D.Y., Ding S.Y., Lin H.X., Liu B.J., Ye Z.Z., Fan F.R., Ren B., Tian Z.Q. (2012). Distinctive enhanced and tunable plasmon resonant absorption from controllable Au@Cu_2_O nanoparticles: Experimental and theoretical modeling. J. Phys. Chem. C.

[19] Cortés-Juan F., Ramos C.C., Connolly J.P., David C., García de Abajo F.J., Hurtado J., Mihailetchi V.D., Ponce-Alcántara S., Sánchez G. (2013). Effect of Ag nanoparticles integrated within antireflection coatings for solar cells. J. Renew. Sustain. Energy.

[20] Beck F.J., Polman A., Catchpole K.R. (2009). Tunable light trapping for solar cells using localized surface plasmons. J. Appl. Phys..

[21] Ouyang Z., Zhao X., Varlamov S., Tao Y., Wong J., Pillai S. (2011). Nanoparticle-enhanced light trapping in thin-film silicon solar cells. Prog. Photovolt. Res. Appl..

